# Individual differences in emotional reactivity: A multidimensional systematic review across the big five

**DOI:** 10.1371/journal.pone.0357470

**Published:** 2026-09-09

**Authors:** Dung Ngoc Nguyen, Chi Le Phuong Trinh, Son Van Le Phan, Oanh Tran Yen Pham, Ngan Thi Thanh Nguyen

**Affiliations:** 1 KU Leuven, Leuven, Belgium; 2 University of Social Sciences and Humanities, Vietnam National University Ho Chi Minh City, Vietnam; 3 International Psychoanalytic University Berlin, Germany; James Cook University, SINGAPORE

## Abstract

**Background:**

Emotional reactivity reflects how individuals respond to emotional stimuli and can be characterized by multiple dimensions, including intensity, sensitivity, and persistence. Together with dispositional tendencies toward positive and negative emotional experiences, these dimensions define an individual’s emotional profile. Despite substantial research, evidence remains fragmented, with a predominant emphasis on Neuroticism and negative emotions, hindering a comprehensive understanding of emotional reactivity across the Big Five personality traits.

**Methods:**

This systematic review synthesized evidence on how the Big Five personality traits relate to core domains of emotional reactivity, including intensity, sensitivity, persistence, and the frequency of positive and negative emotional experiences. A total of thirty records, comprising 31 studies published between 1999 and 2024 and yielding 50 independent samples, were included and analyzed using a narrative synthesis approach. The study protocol was preregistered on PROSPERO under CRD420251067878.

**Results:**

Personality traits showed distinct patterns of emotional reactivity across multiple domains. Neuroticism displayed the most consistently maladaptive profile, characterized by heightened negative affect, greater sensitivity to stressors, and prolonged emotional recovery. Extraversion and Conscientiousness were generally associated with more adaptive emotional profiles, particularly greater positive emotionality. Openness to Experience showed a more heterogeneous profile, including heightened sensitivity and divergence between subjective and physiological responses, whereas Agreeableness reflected a predominantly prosocial profile with context-dependent variation in negative emotional responses.

**Conclusions:**

The findings support a multidimensional conceptualization of emotional reactivity across the Big Five personality traits. This perspective may improve understanding of individual differences in emotional functioning and mental health outcomes.

## Introduction

### Emotion and Individual Differences

Although the precise psychological structure of emotions remains a subject of ongoing theoretical debate, emotions are generally understood as biologically grounded, relatively short-term responses to internal and external stimuli [1]. Alongside other cognitive processes such as perception and memory, emotions play a fundamental role in organizing and conveying meaning within the continuous stream of human experience. They reflect how individuals appraise and respond to both external stimuli, such as objects, people, or events, and internal stimuli, including their own thoughts [2]. These responses are inherently complex, involving the coordinated interplay of multiple components, including physiological arousal, cognitive appraisal, expressive behavior, and motivational tendencies [1]. Importantly, these integrated mechanisms are not rigid but dynamic and flexible, enabling individuals to adaptively shape their engagement with unfolding situations [3]. This multifaceted nature makes emotions a powerful force that supports a wide range of functions, from ensuring survival to facilitating communication and strengthening social bonds [2].

Although the functions of emotions are shared across human beings, individuals display distinct patterns of emotional reaction, which can be described as affective style [4,5]. Such variability is partly explained by differences in appraisal processes, whereby the same event can elicit divergent emotional responses depending on how it is interpreted [6]. Beyond cognitive appraisal, research has identified a range of more fundamental contributors to these differences, including personality, temperament, dispositional affect, biological sex, genetic factors, and environmental influences [2,7–9]. Together, these interacting factors contribute to the development of relatively stable emotional profiles that guide how individuals respond to their environments across the lifespan [10].

To characterize variation in emotional experiences, research has focused on several core characteristics of emotional responses, including intensity, sensitivity, duration to emotional stimuli, and dispositional tendencies toward positive or negative emotionality. In particular, some individuals experience more intense and longer-lasting emotions, whereas others exhibit more moderate reactions to salient events [11]. This variation extends to sensitivity with some individuals showing heightened responsiveness to emotional stimuli. For example, carriers of the short allele of the serotonin transporter gene (5-HTTLPR) show heightened attentional responses to emotional cues, particularly threats [12], and in some cases even positive stimuli such as happy faces [13–15]. Over time, repeated exposure to emotionally salient stressors may lower the threshold for emotional activation, contributing to a relatively enduring state of heightened sensitivity [16,17]. Additionally, stable dispositional tendencies influence the extent to which individuals experience recurrent and intense positive emotional states, or are vulnerable to negative emotionality marked by chronic patterns of anxiety, sadness, and psychological distress [11].

Individual differences in emotional profiles carry significant implications for physical health, mental well-being, career trajectories, and social relationships. Individuals who are prone to fear, shame, or social-evaluative threat often exhibit elevated cortisol and inflammatory responses, increasing their risk for adverse physical health outcomes [18–21]. In contrast, frequent experiences of positive emotions have been linked to reduced inflammation, indicating a potential biological mechanism underlying improved overall health [22]. Emotional instability and heightened fluctuations in affective intensity are also associated with psychological disorders, including depression, anxiety, and borderline personality disorder [23]. In the professional domain, individuals who consistently display assertive and socially engaged behaviors are more likely to attain higher status in the workplace and community contexts, contributing to long-term income growth [24–26]. Similarly, emotional tendencies shape the quality of close relationships, as heightened sensitivity to rejection or threat, along with expressions of tension and distress, can undermine trust and relational stability [27,28].

### The Big Five Personality and Emotional Differences

Among the various frameworks proposed to explain individual differences in emotional experience, personality models offer a particularly appropriate integrative perspective. Although individual emotional reactions are typically transient, consistent tendencies to experience particular emotions over time may become characteristic aspects of an individual’s personality [2]. Moreover, personality traits have been conceptualized as enduring dispositions that systematically organize patterns of affect, cognition, and behavior [29]. Because emotional responses emerge through the interaction of these processes [30], personality can also provide a theoretically grounded framework for understanding how and why individuals experience emotions in distinctive ways.

Among the numerous theoretical frameworks proposed to explain the structure of personality, the Five Factor Model (FFM) has emerged as one of the most influential and empirically supported models in contemporary psychology [31]. Compared to earlier personality theories, the FFM provides a parsimonious, hierarchical structure encompassing both broad traits and narrower facets, demonstrates strong cross-informant validity, and shows robust predictive power across diverse life domains [32]. The model organizes personality into five broad dimensions: Openness to Experience, Conscientiousness, Extraversion, Agreeableness, and Neuroticism [33]. *Openness to Experience* reflects curiosity, imagination, and receptivity to novelty and complexity in thoughts and experiences. *Extraversion* encompasses stimulation-seeking, sociability, enthusiasm, and positive emotionality, shaping individuals’ engagement in dynamic social environments. *Conscientiousness* includes self-discipline, organization, and reliability, supporting goal pursuit and responsible behavior. *Agreeableness* is characterized by empathy, trust, and cooperation, facilitating harmonious and prosocial relationships. In contrast, *Neuroticism* reflects a predisposition toward negative emotionality, including anxiety, anger, and emotional instability, which can contribute to interpersonal problems.

Recent work has further reinforced the utility of the Five-Factor Model as a higher-order framework for organizing personality constructs relevant to emotional differences. For example, while Reinforcement Sensitivity Theory (RST) explains individual differences in emotional and motivational responding through systems related to reward, punishment, and threat, Alblas et al. [34] demonstrated that RST scales can be meaningfully located within the Big Five trait space. Beyond this theoretical support, extensive empirical research has directly demonstrated that the Big Five personality traits are systematically associated with individual differences in emotional experience. *Neuroticism* shows the strongest and most consistent associations with negative emotions, such that individuals high in Neuroticism experience anxiety, fear, and self-disgust more frequently, intensely, and for longer durations, often accompanied by heightened emotional reactivity and less effective regulation [35–39]. In contrast, *Extraversion* is strongly linked to positive emotions such as joy, pride, and happiness; extraverted individuals tend to experience these emotions more frequently and sustain them over time [36,40,41]. Although findings regarding reactivity to positive stimuli are mixed, extraverts generally show faster recovery from stress and lower intensity of negative emotions [38,39,42].

*Openness* has been associated with specific positive emotions such as compassion, joy, and love, particularly in contexts involving intellectual or emotional complexity, and may predict heightened reactivity to positively valenced experiences [39,41]. Similarly, *Agreeablenes*s is linked to socially oriented positive emotions and greater peak emotional intensity, though not necessarily to faster emotional responses; whereas lower Agreeableness is associated with hostility and antagonistic affect [36,41,43]. Finally, *Conscientiousness* relates to self-regulated positive emotions such as attentiveness and pride, particularly in goal-directed contexts, and is associated with lower stress and fear, reflecting effective emotional control rather than heightened emotional reactivity [35,36,41]. Collectively, these findings support the use of the Big Five as an integrative framework for synthesizing evidence on individual differences in emotional experiences. However, existing conceptualizations of how personality traits are expressed emotionally have largely emphasized valence, often characterizing traits in terms of positive versus negative tendencies. As a result, personality-related differences in emotional experiences have not been systematically synthesized from a broader multidimensional perspective.

### Emotional reactivity

Individual differences in emotional experiences encompass a range of characteristics and processes. To provide a focused yet multidimensional synthesis of the emotional profiles associated with the Big Five personality traits, this review adopts the framework of emotional reactivity, which captures core aspects of how individuals respond to emotionally evocative stimuli. In particular, emotional reactivity refers to the degree to which individuals differ across three interrelated dimensions: sensitivity (responsiveness to emotional stimuli), intensity (the magnitude of emotional responses), and persistence (the duration of emotional activation before returning to baseline; [44]). For conceptual clarity, emotional reactivity in the present review is distinguished from some related emotional constructs, such as emotional awareness (the ability to conceptualize and describe one’s own emotions and those of others), emotion regulation (attempts to influence which emotions one has and how those emotions are experienced or expressed), and emotional expression (the behavioral changes associated with emotion) [45–47]. Within this review, emotional reactivity refers specifically to individual differences in the sensitivity, intensity, and persistence of emotional responses to emotionally evocative stimuli. Importantly, emotional reactivity is also valence-specific, as individuals may respond differently to positive and negative stimuli. For example, neurotic individuals show heightened emotional reactivity to negative-mood inductions while extraverts show greater reactivity to positive inductions [48]. In clinical populations, individuals with major depressive disorder display reduced emotional reactivity to both negative and positive stimuli, with a more pronounced reduction for positive stimuli [49].

Accordingly, measurement approaches have evolved to capture this multidimensional and valence-sensitive structure using complementary methodologies. The Emotion Reactivity Scale (ERS; [44]) assesses subjective sensitivity, intensity, and persistence and demonstrates strong internal consistency. To incorporate valence specificity, the Perth Emotional Reactivity Scale (PERS) was later developed to measure these components separately for positive and negative emotions [50]. Beyond self-report, physiological research examines the temporal dynamics of reactivity through affective chronometry [51]. Within this framework, sensitivity and intensity are typically indexed by autonomic arousal (e.g., heart rate, skin conductance, respiratory sinus arrhythmia) in response to standardized stimuli, whereas persistence is operationalized as recovery time, reflected in the return of cortisol or cardiovascular markers to baseline following stress [52]. Neuroimaging studies further suggest distinct neural correlates of reactivity components, including heightened cingulate and premotor activation linked to sensitivity, increased ventral tegmental area responses associated with positive intensity, and sustained amygdala activity indicative of persistence [49,53].

Emotional reactivity holds substantial clinical relevance and has been identified as a transdiagnostic vulnerability factor across psychiatric conditions [54]. Elevated reactivity is associated with mood, anxiety, and eating disorders and mediates the link between psychopathology and self-injurious thoughts and behaviors [44]. Heightened reactivity also predicts depressive symptoms and suicidal ideation [55], contributes to health anxiety [56], and functions as a vulnerability marker for psychotic illness [57]. Collectively, these findings highlight emotional reactivity as a central mechanism through which personality traits may shape risk for emotional dysfunction and psychopathology, thereby motivating further research in this area.

### Research gap and study objectives

Emotional reactivity has frequently been examined within the broader domain of personality [44], particularly in relation to Neuroticism. Indeed, the most consistent empirical evidence links high Neuroticism (often conceptualized as negative emotionality) to heightened emotional reactivity, whether assessed through neural indicators or daily affective fluctuations [58–60]. However, although emotional reactivity and personality are conceptually related, they remain theoretically and functionally distinct constructs. Personality reflects a broad constellation of enduring individual differences, encompassing cognitive styles, behavioral tendencies, motivational orientations, interpersonal functioning, and self-regulatory capacities [32,33]. By contrast, emotional reactivity refers to a more specific aspect of emotional functioning, concerning individual differences in how readily emotional responses are elicited, how strongly they are experienced, and how long they persist following emotionally relevant stimuli [44]. Yet, in practice, Neuroticism is often treated as a proxy for emotional reactivity, thereby blurring conceptual boundaries and obscuring finer-grained components such as sensitivity (threshold), intensity (arousal magnitude), and persistence (duration of activation). This conflation limits theoretical clarity and hinders precise mapping between trait structure and emotional processes.

Moreover, the existing literature has predominantly examined emotional reactivity within negative emotional contexts, particularly in association with Neuroticism. This narrow focus leaves important questions unresolved, including whether heightened reactivity associated with Neuroticism is confined to negative affect or reflects a broader pattern of emotional responsiveness. At the same time, the uneven coverage of the remaining Big Five traits (i.e., Extraversion, Agreeableness, Openness, and Conscientiousness) underscores the need for a broader and more integrative synthesis. Although quantitative reviews have examined related constructs such as emotion regulation strategies (e.g., [31,61]), no systematic review to date has comprehensively synthesized evidence on emotional reactivity across all five personality dimensions. As a result, our understanding of how personality shapes emotional functioning at the level of reactivity, a foundational process that precedes regulation, remains fragmented.

Accordingly, the present systematic review examined how the Big Five personality traits are associated with different dimensions of emotional reactivity, including intensity, sensitivity persistence, and the tendency to experience positive versus negative emotions. These predefined domains served as the framework for the narrative synthesis, enabling consistent comparisons across heterogeneous studies and facilitating the identification of trait-specific patterns of emotional reactivity. Specifically, the review aimed to (1) synthesize evidence on the associations between the Big Five personality traits and different dimensions of emotional reactivity, (2) identify patterns, consistencies, and inconsistencies across the literature, and (3) provide a more integrated conceptual synthesis of personality-related differences in emotional reactivity. In addition, the review identifies gaps in the existing literature and discusses the implications of these findings for understanding the relationships between personality, emotional reactivity, and well-being.

## Materials and methods

This systematic review was conducted following the Preferred Reporting Items for Systematic Reviews and Meta-Analyses (PRISMA) guidelines [62]. In line with the PRISMA Checklist, the study protocol was preregistered on the PROSPERO.

### Eligibility criteria

To be included in this review, studies had to meet the following criteria: (1) be empirical studies in nature and employ quantitative methods, including cross-sectional, longitudinal, or experimental designs; (2) assess at least one of the Big Five personality traits alongside at least one indicator of emotional reactivity; and (3) report at least one direct association between personality traits and emotional processes. To ensure the quality and accessibility of evidence, only peer-reviewed articles published in English with full-text availability were included in the final synthesis. The detailed inclusion and exclusion criteria are summarized in Table 1.

**Table 1:** Overview of the inclusion and exclusion criteria used in the screening process.

	Inclusion criteria	Exclusion criteria
**Study design**	Quantitative Cross-sectional Longitudinal Experimental	Qualitative Case study Commentary Review Theoretical papers
**Comparison and outcomes**	Reliable measure of personality traits Reliable measure of emotional mechanisms Association between personality and emotional mechanisms	Unreliable measure of personality traits Unreliable measure of emotional mechanisms No association between personality and emotional mechanisms
**Publication status**	Published in English Peer-reviewed Full-text accessibility	Unpublished Preprints Non-peer-reviewed

### Types of participants

No restrictions were applied regarding participants’ age, gender, cultural background, or clinical status. Studies involving both clinical and non-clinical samples, across all age groups and nationalities, were eligible for inclusion. This inclusive approach was intended to capture a comprehensive picture of how emotional processes vary across individuals.

### Types of comparison and outcome measures

Studies were eligible for inclusion if they reported statistical data on the association between Big-Five personality traits and emotional reactivity, operationalized primarily through components such as arousal (or intensity), persistence, sensitivity, negativity, and positivity. To ensure consistency in measurement, only studies that used validated instruments to assess both personality and emotional processes were included.

(1) For personality assessment, eligible studies were required to assess at least one Big Five personality trait using standardized personality inventories with established evidence of reliability and validity, such as the NEO Personality Inventory-Revised (NEO-PI-R) or the Big Five Inventory (BFI).
(2) Regarding emotional reactivity, eligible studies were required to assess at least one dimension of emotional reactivity (i.e., intensity, persistence, or sensitivity), together with the frequency of positive or negative emotions as a complementary dimension, using measures with established evidence of reliability and validity, including standardized psychological scales as well as neural and physiological indicators of emotional responding.

### Search Strategy

A comprehensive literature search was conducted across four major databases including PsycARTICLES, PubMed, Web of Science, and Scopus, which cover all publication years up to May 30, 2026. Search terms were organized into two primary conceptual categories: Personality and Emotion Reactivity, and were applied systematically across databases. Given that emotion reactivity is a multifaceted construct encompassing diverse definitions and processes, the search strategy was broadened to capture this complexity. In addition to the core components of intensity, persistence, and sensitivity, related terms such as duration, activation, response, dynamics, variability, instability, state, experience, and lability were also included to ensure comprehensive coverage of relevant literature (see Table 2 for full details).

**Table 2:** A summary of search terms across all databases.

Concept	Construct	Search terms
**Personality**	A. Personality	Big Five Five Factor Model FFM Personality Traits Neuroticism Extraversion Agreeableness Conscientiousness Openness
**Emotion Reactivity**	B. Emotion	Emotion* Affect*
	C. Emotional Reactivity Characteristics D. Positivity/Negativity (Valence)	Reactivity Arousal Intensity Persistence Duration Sensitivity Activation Response* Responsiveness Dynamic* Variability Instability State Experience Lability

***Note:*** *Final Search =* ***(A)*** *AND* ***(B*** *AND (****C*** *OR* ***D)).***

### Selection of Studies

**Fig 1:**
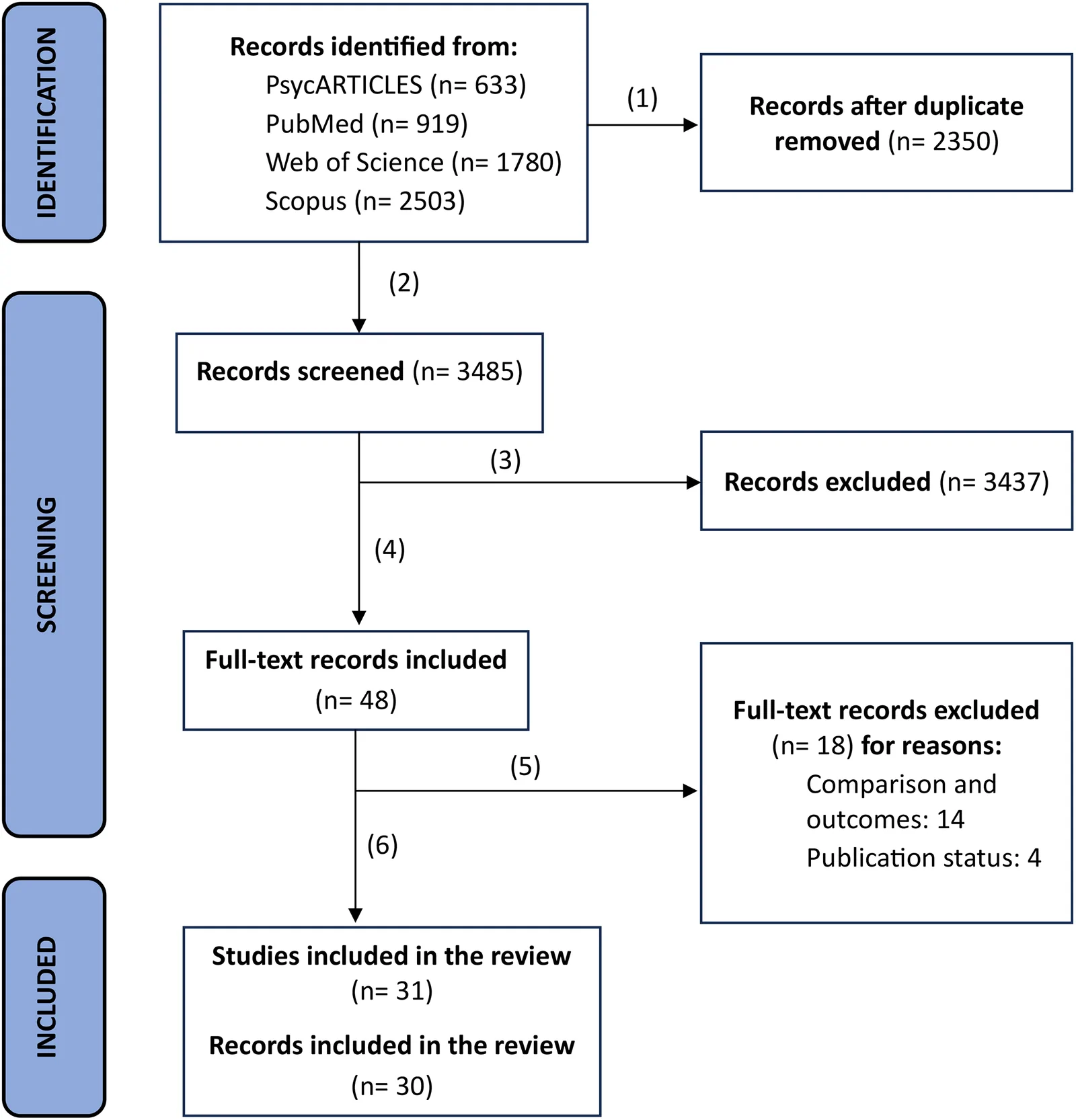
PRISMA flow diagram of selecting studies to be included in the systematic review.

Two independent reviewers were involved at every stage to ensure methodological rigor and reduce bias. First, all identified records were exported into reference management software. Duplicate entries were removed prior to screening. Titles and abstracts of the remaining articles were screened independently by both reviewers using Rayyan, a web-based tool designed to facilitate blinded and collaborative screening of studies. Following the initial screening, the full texts of potentially eligible articles were retrieved and assessed independently by the two reviewers. Disagreements regarding eligibility were resolved through discussion until consensus was reached. The flow of studies through the identification, screening, and inclusion stages is illustrated in the PRISMA flow diagram (see Fig 1) and checklist (S1 Checklist).

### Quality Assessment

To evaluate the methodological quality of the included studies, we used the Joanna Briggs Institute (JBI) Critical Appraisal Tools (https://jbi.global/critical-appraisal-tools) as a foundation. These tools provide structured checklists tailored to various study designs and are widely used for assessing the risk of bias in quantitative research. Given the methodological diversity of the studies included in this review (e.g., cross-sectional, longitudinal, experimental designs), we developed two customized appraisal forms based on combinations of relevant JBI checklists. For observational studies (cross-sectional and longitudinal), a single unified checklist was created by integrating items from the JBI Checklist for Analytical Cross-Sectional Studies and the Checklist for Cohort Studies. For experimental studies, a second checklist was adapted by combining items from the JBI Checklists for Randomized Controlled Trials (RCTs) and Quasi-Experimental Studies. (Note: if no quasi-experimental designs are included after data extraction, we default to using the original JBI checklist for RCTs alone). Each criterion in these tools was evaluated as “Yes,” “No,” “Unclear,” or “Not Applicable” based on explicit guidance to ensure transparency and inter-rater consistency. Two independent reviewers conducted the quality appraisal for each study. In cases of disagreement, discrepancies were resolved through discussion. Rather than numerical scores, assessments were used descriptively and informed sensitivity analyses during synthesis. Full details of the appraisal criteria and adapted checklists are provided in S2 Table (JBI Quality Assessment results). No overall quality score or cutoff threshold was applied to define study quality or determine study eligibility; instead, all studies were appraised across domains and interpreted qualitatively within the narrative synthesis.

### Data extraction and synthesis

Eligibility criteria and study selection were guided by the PICOS framework [63]. Based on these predefined criteria, the full texts of potentially eligible studies were downloaded and independently reviewed by two authors. The studies included in the review were selected based on predefined criteria, including the publication year, language, study design, and relevance to the Big Five personality traits and emotional reactivity. Any studies that did not meet these criteria were excluded from the analysis. To ensure consistency in data extraction, the two authors independently reviewed the papers, resolved any disagreements through discussion, and a senior author conducted an informal assessment of the review process to evaluate the appropriateness of the extracted data.

The collected information was then organized into Table 5, which includes the following categories: (1) publication characteristics, such as the publication year, country of data collection, and authors; (2) sample characteristics, including the total sample size, gender composition, mean age with standard deviation, and participants’ clinical or health states; (3) methodological details, covering the type of research design and the instruments used to assess Big Five personality traits and emotion reactivity; and (4) main results, outlining the associations among the phenomena of interest. When a publication reported multiple independent studies or samples, each was treated as a separate unit of analysis during data extraction and narrative synthesis.

Given the conceptual diversity of emotional processes and personality traits across studies, extracted variables were grouped into key domains to facilitate comparison and synthesis. Personality traits were categorized according to the Big Five model, comprising Neuroticism, Extraversion, Openness to Experience, Agreeableness, and Conscientiousness. When studies reported multiple traits, each was coded separately. For emotion reactivity, variables were classified into three domains: sensitivity, intensity, persistence. These categorizations were informed by existing theoretical frameworks (e.g., [44]) to support clearer cross-study comparisons. In addition, the frequency of valence (i.e., positivity and negativity) was examined as a complementary dimension. Due to the heterogeneity of study designs and outcome measures, a meta-analysis was not conducted. Instead, a narrative synthesis approach was employed to summarize and interpret the quantitative evidence across the included studies.

To further address conceptual and methodological variability in the operationalization of emotional processes, a coding scheme was developed (Table 3) to standardize the classification of emotional outcomes. This scheme delineates core domains of emotional functioning, each defined by a guiding question, conceptual scope, and typical indicators. It was applied to systematically extract and organize findings across studies, enabling a more consistent and theoretically grounded synthesis. By clarifying how different measures map onto specific emotional domains, the scheme enhances comparability across studies and strengthens the interpretability of the review. Finally, it is important to note that the term emotion is used as an umbrella construct encompassing both positive and negative emotional experiences, including those labeled as affect in the original studies. Terminology from primary studies is retained where necessary for accuracy but synthesized within a unified emotional framework.

**Table 3:** Coding scheme and descriptive criteria.

Domains of Emotion	Core Question	Conceptual Focus	Examples of Typical Indicators
**1. Intensity**	How strong is the emotional response when it occurs?	Magnitude of emotional activation	- Self-reported emotion intensity (e.g., “How intense did you feel?”) - Physiological amplitude (e.g., EDA, heart rate increase) - Peak levels during emotional events
**2. Sensitivity**	How easily is an emotional response triggered?	Threshold for emotional activation	- Reactivity to mild or ambiguous stimuli - Faster response latency - Greater likelihood of responding - Interpersonal or environmental sensitivity, magnitude of affective change in response to contextual input (e.g., hassles vs. no hassles).
**3. Persistence**	How long does the emotion last once triggered?	Emotional recovery and decay over time	- Time to return to baseline - Emotional inertia - Prolonged negative emotions - Slower physiological recovery / habituation - Tendency to savor specific emotions
**4. Frequency (Positivity/ Negativity)**	How often and to what extent are positive versus negative emotions experienced?	Overall levels and relative balance of positive and negative emotional experiences across time	- Mean levels of positive and negative emotions - Proportion of positive relative to negative emotions - Frequency of positive and negative emotions

## Results

A concise overview of the main findings is presented in Table 4. A comprehensive summary of all extracted findings is provided in Table 5.

**Table 4:** Summary of Big Five Personality Traits and Emotional Reactivity Domains.

Personality Trait	Frequency	Intensity	Sensitivity	Persistence
**Openness to Experience**	More frequent positive emotions [64–69]; mixed findings for negative emotions [36,64–66,70–72].	Lower subjective negative intensity but higher physiological responses [73,74]; positive intensity findings are mixed [68,69,73].	Higher sensitivity to aesthetic, social–emotional, and musical stimuli [75,76].	*(Not examined in eligible studies)*
**Agreeableness**	More frequent prosocial positive emotions [36,66,67,72,77]; generally lower negative emotions [66,68,71,78], with context-dependent variation [79–81].	Greater positive emotional intensity [69,73], lower anger intensity [78], stronger negative affect intensity after sadness induction [73].	Higher aesthetic and social sensitivity to environmental and interpersonal cues [75]; greater stress reactivity to daily hassles [82].	Shorter persistence of negative emotions [78].
**Conscientiousness**	More frequent positive emotions [36,66,68]; less frequent negative emotions [36,66,81,83], including on stressor days [77].	Lower subjective negative intensity [68,84] but higher physiological activation [74]; greater positive intensity in social contexts [69].	Lower subjective [75] but higher physiological sensitivity to negative stimuli [85].	(Not examined in eligible studies)
**Neuroticism**	More frequent negative emotions and reduced positive emotions [36,64–66,68,71,72,77,79–81,84,86,87].	Higher self-report negative affect intensity [73] and, in some studies, higher self-report positive affect intensity [73]; greater physiological arousal to aversive stimuli [74,88].	Greater sensitivity to stressors and daily hassles [17,77,82,89].	Prolonged negative emotions, poorer recovery, and reduced maintenance of positive emotions [37,38,88].
**Extraversion**	More frequent positive emotions [36,64,65,68,77,80]; generally fewer negative emotions [36,64,65,68,77,80] with less consistent evidence [66,72,79].	Higher self-report positive emotional intensity [38,73]; lower physiological reactivity to threatening stimuli [74].	Greater responsiveness to music-evoked emotions [76].	Longer persistence of positive emotions [38], potentially supported by greater savoring [37].

**Table 5:** A comprehensive summary of all extracted findings.

Author (year)	Country	Sample typology	N (F/M)	Age in years M (SD)	Design	Personality Assessment	Affective Assessment	Main Results	Dimension
1. Verduyn & Brans (2012) [38]	Belgium	Community	98 (61/37)	19 (1.3)	Longitudinal	Ten Item Personality Inventory (Gosling, Rentfrow, & Swann, 2003) Dutch version (Hofmans, Kuppens, & Allik, 2008)	Daily questionnaire about frequency, intensity and duration of emotion episodes (Verduyn, Delvaux et al. 2009 & Verduyn et al. 2011): positive and negative clusters	Higher (E) was associated with greater frequency, intensity, and duration of positive emotions, with duration showing the strongest association. Higher (N) was associated with greater frequency and duration of negative emotions, with frequency showing the strongest association.	Frequency (Positivity/Negativity) Intensity Persistence
2. Leger et al. (2016) [77] Study 1	US	Community	1750	57	Longitudinal	MIDUS Big Five Scale (Prenda & Lachman, 2001).	Daily negative affect in NSDE II (13 negative emotions) Daily positive affect in NSDE II (13 positive emotions)	Higher (C), (O), (A), and (E) were associated with higher daily positive affect and lower daily negative affect. Higher (N) was associated with lower daily positive affect and higher daily negative affect.	Frequency (Positivity/Negativity)
3. Jensen-Campbell et al. (2007) [83]	US	Community	126 (63/63)	21.24 (4.03)	Experimental	Big Five Inventory (John et al., 1991) Trait Markers (Goldberg, 1992): 100 trait words	Rating 24 emotions on a Likert scale 1–5 (Watson et al.,1988)	Higher (C) was associated with lower anger. Lower (A) was associated with higher anger, particularly among individuals low in (C).	Frequency (Negativity) Intensity
4. Hepp et al. (2016) [84]	US	Clinical	114 (114/0)	BDP (N = 74): 32.4 (11.9) MDD and/or DD (n = 40): 34.8 (12.2)	Longitudinal	NEO personality inventory revised (NEO PI-R; Costa & McCrae, 1992)	Negative affect instability: quantified by squared successive differences Positive and Negative Affect Schedule-Extended version (PANAS-X; Watson & Clark, 1999). Using 21 negative affect items.	Higher (C) was associated with lower negative affect in the BPD group. Higher (N) was associated with higher negative affect in both the BPD and depressive groups.	Intensity
5. Roesch et al. (2009) [70]	US	Community	673	19.86 (2.70)	Longitudinal	NEO-FFI (Costa & McCrae, 1989)	20-item Naval Health Research Center Mood Questionnaire	Higher (N) and (O) were associated with higher negative affect. Higher (C) was associated with higher positive affect.	Frequency (Positivity/Negativity)
6. Bresin et al. (2012) [78]	US	Community	Study 1: 94 (62 F) Study 2: 129 (77 F) Study 3: 96 (69/32)	Not mentioned	Study 1: cross-sectional Study 2&3: Longitudinal	Study 1 &2: 10-item broad-bandwithscale measuring Agreeableness (Goldberg, 1999) Study 3: 20 unipolar markers for agreeableness (Goldberg, 1992)	Study 1&2: Subscale Anger (Watson & Clark, 1994) Study 3: Measure the percentage of waking time spent experiencing anger on an 8-point scale	Higher (A) was associated with lower anger intensity under high physiological arousal. Higher (A) was also associated with shorter persistence of anger in daily life.	Intensity Persistence
7. van Oosterhaut et al. (2022) [86]	Netherlands	Clinical	ASD: 50 (24/26) Non-ASD: 51 (25/26)	ASD: 41.1 (12.9) Non-ASD: 35.5 (12.2)	Cross-sectional	12-item Subscale Neuroticism (Dutch version NEO Five-Factor Inventory; Hoekstra et al., 2007)	Negative Affect assessment: Likert scale 7-point, adj: insecure, lonely, down, anxious Momentary stress assessment: activity-related stress, event-related stress, social stress	In the ASD group, higher (N) was associated with higher momentary negative affect. Higher (N) was also associated with stronger negative affect in socially unfamiliar contexts and under activity/social stress.	Frequency (Negativity)
8. Rolland & Fruyt (2003) [81]	France	Community (Male military)	Time 1: 460 Time 2: 130 (All M)	Time 1: 26.34 (6.55) Time 2: 27.99 (7.34)	Longitudinal 6-month interval between Time 1 and Time 2.	Big Five Adjective self-report list (Rolland, 1993; Rolland & Mogenet, 2001): 5 dimensions, 11 adj each.	Affects experienced at work (based on Diener et al., 1995): frequency of 4 emotions: Anger, Fear, Sadness, Shame.	Higher (N) was associated with higher levels of anger, fear, sadness, and shame at both Time 1 and Time 2. Higher (A) was associated with higher fear at both time points. Higher (C) was associated with lower shame at Time 1 only.	Frequency (Negativity) Persistence
9. Griffin et al (2006) [87]	US	Community (96% veterans)	1534 (All M)	M = 69 (SD not stated)	Longitudinal	EPI-Q by Eysenck Personality Inventory (Eysenck & Eysenck, 1968): assessing Extraversion and Neuroticism	Positive and Negative Affect by PANAS (Watson et al., 1988)	Higher (E) was associated with higher positive affect levels, but not with change in positive affect over time. Higher (N) was associated with higher negative affect and with less midlife decline and stronger later-life increases in negative affect.	Frequency (Positivity/Negativity)
10. Mroczek et al (2004) [17]	US	Community	1012 (551/461)	47.95 (13.15)	Cross-sectional	Neuroticism (Goldberg, 1992)	Daily negative affect assessed by Item Response Theory (Kessler et al., 2002)	Higher (N) was associated with higher daily negative affect.	Frequency (Negativity)
11. Vogeltanz, N. D., & Hecker, J. E. (1999) [88]	US	Community	Time 1: 200 Time 2: 94 (All F)	Not provided	Experimental	Neuroticism by Eysenck Personality Questionnaire (EPQ; Eysenck & Eysenck, 1975)	Physiological reactivity: the frequency of spontaneous skin conductance responses Subjective arousal: self-report rating scale from 0 (very low) to 8 (very high)	Higher (N) was associated with greater physiological and subjective arousal to aversive stimuli and with slower physiological habituation.	Intensity Persistence
12. Weiting Ng &Ed Diener (2009) [37]	US	Community	Study 1: 144 Study 2: 236 F/M not specified	Not provided	Experimental	Neuroticism and Extraversion: International Personality Item Pool (IPIP; Goldberg et al., 2006)	Positive and negative emotions (Self-report intensity ratings of emotional interaction on 7-point scale)	Higher (N) was associated with poorer reduction of negative emotion when negative stimuli diminished. Higher (N) was associated with poorer maintenance of positive emotions. Higher (E) was associated with better maintenance of positive emotions.	Persistence
13. Fuente et al. (2024) [80]	Spain	Community	637 (535/91)	21.33 (SD not provided)	Cross-sectional	Big Five Questionnaire (Barbaranelli et al., 2003)	Positive and Negative Affect Scale (PANAS-N)	Higher (C), (E), (O), and (A) were associated with higher positive affect, although the associations for (O) and (A) were weaker. Higher (E) and (C) were associated with lower negative affect. Higher (A) and (N) were associated with higher negative affect, with (N) showing the strongest association.	Frequency (Positivity/Negativity)
14. Deak et al. (2024) [67]	Hungary	Community	240 (177/63)	27.84 (12)	Cross-sectional	(Big Five Inventory, BFI – Hungarian version, 44-item scale)	Positive (SEEK, PLAY, CARE) and negative (ANGER, SADNESS, FEAR) emotional dimensions through the Affective Neuroscience Personality Scales	Higher (A) was associated with higher CARE and lower ANGER. Higher (E) was associated with higher SEEK, PLAY, and ANGER, and with lower FEAR. Higher (C) was associated with higher SEEK and CARE, and with lower FEAR, ANGER, PLAY, and SADNESS. Higher (O) was associated with higher SEEK.	Frequency (Positivity/Negativity)
15. Yang & Koo (2022) [71]	Taiwan	Community	580 (460/120)	21.3 (1)	Cross-sectional	Big Five Inventory (John et al., 1991)	Negative emotional states were evaluated with DASS-21	Higher (N) and (O) were associated with higher negative emotional states. Higher (A) was associated with lower negative emotional states.	Frequency (Negativity)
16. Pekrun et al. (2023) [66] Study 2	US	Community	235 (131/104)	19.36 (1.35)	Prospective/Longitudinal (8-week interval)	NEO-FFI (Costa & McCrae, 1992)	Achievement Emotions Questionnaire – Revised (AEQ; Pekrun et al., 2011)	Higher (N) was associated with higher negative achievement emotions and lower positive achievement emotions. Higher (E) was associated with pride, but also with anxiety, shame, and disappointment. Higher (O) was associated with lower anxiety. Higher (C) was associated with higher positive and lower negative achievement emotions. Higher (A) was associated with hope and relief, and with lower boredom.	Frequency (Positivity/Negativity)
17. Sivathasan, S., Philibert-Lignières, G., & Quintin, E.-M. (2021) [76]	Canada	Community	110 (74/36)	21.25 (3.36)	Cross-sectional	(NEO-FFI-3-Short Form) (McCrae & Costa, 2010)	Gold-MSI-Emotion (Müllensiefen et al., 2014)	Higher (E) was associated with greater emotional responsiveness to music. Higher (O) was associated with greater emotional responsiveness to music.	Sensitivity
18. Klaiber et al. (2022) [69]	US	Community	NSDE 2: 1,919 (1,113/806) NSDE Refresher: 778 (428/350)	NSDE 2: 35–84 years NSDE Refresher: 25–75 years	Correlational Longitudinal (daily diary for 8 consecutive days)	Midlife Development Inventory Personality Scale (Lachman & Weaver, 1997)	Daily affect: 13-item positive and 14-item negative affect scales (0–4 frequency; Kessler et al., 2002; Mroczek & Kolarz, 1998). Emotions during positive events (Refresher sample only): Ratings of pleasant, surprised, calm, proud, and close to others (0–3 scale).	Higher (E), (A), (C), and (O) were associated with stronger positive emotional responses during positive events. Higher (N) was associated with lower calmness during positive events.	Intensity
19. Trå et al. (2023) [75]	Norway	Community	Study 1: 1,096 (548/548) Study 2: 328 (164/164)	18-65+ (SD not provided; Majority under 30)	Cross-sectional	Norwegian version of the BFI-44 (Engvik & Føllesdal, 2005; John & Srivastava, 1999)	Highly Sensitive Person scale (Aron & Aron, 1997)	Study 2: Higher (N) was associated with higher sensitivity overall Higher (A) was associated with higher aesthetic and social sensitivity. Higher (O) was associated with higher aesthetic and social-emotional sensitivity. Higher (C) was associated with slightly lower sensitivity.	Sensitivity
20. Martínez et al. (2021) [65]	Spain	Community (Nurse)	1268 (1081/187)	32.02 (6.91)	Cross-sectional	BFI-10 (Rammstedt & John, 2007)	PANAS (Watson et al., 1988)	Higher (E), (A), (C), and (O) were associated with higher positive affect and lower negative affect. Higher (N) was associated with higher negative affect and lower positive affect.	Frequency (Positivity/Negativity)
21. Šoškić et al. (2021) [85]	Serbia	Community	Study 1: 114 (109/5) Study 2: 99 (50/49)	aged 18–25	Cross-sectional	Study 1: NEO–PI–R inventory (Costa, 1992) Study 2: Serbian adaption of HEXACO-100 (Lee & Ashton, 2018) & DELTA-30 (Knezevic et al., 2017)	Study 1: Polyscore (Lafayette-4000 polygraph) Study 2: Electrodermal Activity (EDA)	Higher (C) was associated with greater autonomic response to negatively valenced visual stimuli. In Study 2, higher (C) was associated with stronger electrodermal responding when latency, amplitude, rise time, and reaction presence were analyzed jointly.	Intensity Sensitivity (study 2 only)
22. Wrzus et al. (2021) [82]	Germany	Community	581 (288/293)	42.5 (19.0)	Longitudinal (3 waves: T1 - T2 - T3 in 6 years)	The Big Five Inventory – BFI (BFI; John & Srivastava, 1999; German version: Lang et al., 2011)	Momentary negative affect: Rated (disappointed, downcast, angry, nervous) on 7-point scale (0 = not at all, 6 = very much) Stress reactivity = average NA during hassle moments – average NA during non-hassle moments	Increases in (N) were associated with stronger negative affect reactivity to daily hassles across waves, especially in adolescents and young adults. Increases in (A) were associated with stronger negative affect reactivity to daily hassles only in adolescents and young adults from T1 to T2.	Sensitivity
23. Barford et al. (2020) [89]	Belgium	Community	N = 275 Sample 1: 101 (F: 73.7%) Sample 2: 79 (F: 62.5%) Sample 3: 95 (F: 62.1%)	Sample 1: 21.40 (2.15) Sample 2: 23.5 (7.82) Sample 3: 19.06 (1.28)	Longitudinal (short-term)	Dutch Ten Item Personality Inventory (TIPI) in samples 1 & 3 60‑item NEO‑Five Factor Inventory (NEO‑FFI) in sample 2	Momentary affect (PA, NA): valence-based composites created by averaging arousal-matched positive/negative item pairs (e.g., excited/stressed), rated on 0–100 scales (rescaled to 0–99 in Sample 3)	(N) predicted greater momentary increases in negative affect. (N) was associated with higher average levels of mixed emotional experiences (co-occurrence of positive and negative affect)	Frequency (Negativity)
24. Mill et al. (2016) [64]	Estonia	Community	N = 110 (F: 70/ M: 40)	From 19 to 84 yo Older Group: 68.2 (5.5) Younger group: 21.3 (1)	Experience-sampling (intensive longitudinal) Cross-sectional experience sampling study	Revised NEO Personality Inventory (NEO-PI-R; Costa and McCrae, 1992)	Experience sampling: Positive and Negative Aﬀect Schedule (PANAS; Watson et al., 1988)	Higher (E) was associated with higher retrospective happiness and lower sadness and fear. Higher (N) was associated with lower retrospective happiness and higher sadness, fear, and anger. Higher (C) was associated with lower retrospective sadness, anger, and happiness. Higher (O) was associated with higher retrospective sadness, happiness, and anger. Higher (A) was associated with lower 2-week retrospective happiness.	Frequency (Positivity/ Negativity)
25(a). Letzring & Adamcik (2015) [68] Study 1	US	Community	Study 1: 257 (149/100, Unknown = 8)	Study 1: 23.77 (6.78)	Cross-sectional (Study 1)	Study 1: BFI or IPIP-NEO (domain scales)	PANAS (Watson et al., 1988) Study 1: General affect (“in general”)	Higher (E) was associated with higher positive affect and lower negative affect. Higher (N) was associated with higher negative affect and lower feeling strong. Higher (A) was associated with lower negative affect and slightly higher enthusiasm. Higher (C) and (O) were associated with higher positive affect.	Frequency (Positivity/ Negativity)
25(b). Letzring & Adamcik (2015) [68] Study 2	US	Community	Study 2: 262 (210/89)	Study 2: 24.43 (7.03)	Experimental (Study 2)	Study 2: BFI	Study 2: State affect (“right now”), pre- and post-induction	Higher (E) was associated with greater positive affect reactivity across induction conditions (marginal effect). Higher (C) showed a marginal tendency toward smaller negative affect reactivity under negative induction.	Intensity
26. Ching et al. (2014) [79]	US Venezuela Philippines Japan China	Community	US 56 (33/23) Venezuela 56 (35/21) Philippines 60 (36/24) China (43/23) Japan 54 (28/26)	US 20.32 (3.24) Venezuela 20.16 (1.83) Philippines 17.62 (1.47) China 20.12 (1.34) Japan 20.19 (0.93)	Longitudinal (ESM)	Trait Questionnaire (Church et al., 2008), Big Five trait adjectives, 5-point Likert scale.	Momentary positive affect and negative affect ratings, assessed with four affect adjectives (positive affect: enthusiastic, happy; negative affect: upset, sad), rated on a 5-point Likert scale, based on the circumplex model of affect (Russell, 1980; Watson & Clark, 1994).	Higher (N) was associated with higher negative affect and lower positive affect across cultures. Higher (E) and (A) were associated with higher positive affect across cultures; associations with negative affect were weaker and less consistent. Higher (O) was associated with higher positive affect across cultures. Higher (C) was associated with higher positive affect and lower negative affect only in the Philippines; effects were otherwise weak or non-significant.	Frequency (Positivity/Negativity)
27. Komulainen et al. (2014) [72]	Finland	Community	N = 104 (86/18)	23 (3.69)	Longitudinal	Finnish version of NEO Five Factor Inventory (NEO-FFI)	Self-reported momentary affect ratings Positive and negative emotions rated on a 7-point Likert scale, based on the circumplex model of affect (Russell, 1980).	Higher (N) was associated with lower daily positive affect and higher daily negative affect. Higher (E) was associated with higher daily positive affect. Higher (A) was associated with higher daily positive affect and lower daily negative affect. Higher (C) was associated with lower daily negative affect.	Frequency (Positivity/Negativity)
28. Brumbaugh et al. (2013) [74]	US	Community	169 (101/68)	27 (4.46)	Experimental	Ten-Item Personality Inventory (TIPI; Gos- ling et al. 2003)	Heart rate (HR), skin conductance response (SCR), and respiration (RR) LifeShirt vest for HR & RR ThoughtTechnology GSR sensor for SCR	Higher (N) was associated with higher SCR to violence–fear scenes. Higher (E) was associated with reduced HR to tension and violence–fear scenes. Higher (O) was associated with higher RR during sadness. Higher (C) was associated with higher HR to the gun scene.	Intensity
29. Karreman et al. (2013) [73]	Netherlands	Community	Study 1: 266 (167/99) Study 2: 130 (101/29)	Study 1: 21.93 (2.32) Study 2: 20.12 (2.89)	Study 1: Cross-sectional Study 2: Experimental	Study 1: Dutch 30- item Big Five questionnaire (Gerris et al., 1998) Study 2: NEO-Five Factor Inventory (Dutch NEO-FFI; Hoekstra, Or- mel, & De Fruyt, 1996)	Study 1: Emotional Intensity Scale–Reduced (EIS-R; Geuens & de Pelsmacker, 2002), positive and negative affect intensity subscales, self-report, 5-point Likert scale Study 2: Self-reported ratings of positive and negative emotion intensity, 9-point Likert scale; change scores were used as the reactivity index.	Study 1: Higher (N) was associated with higher positive and negative affect intensity. Higher (A) was associated with higher positive affect intensity. Higher (C) was associated with higher negative affect intensity. Higher (O) was associated with lower negative affect intensity. Study 2 – baseline: Higher (N) was associated with higher baseline negative affect intensity. Higher (E) and (O) were associated with higher baseline positive affect intensity. Study 2 – reactivity: Higher (A) was associated with greater sadness-reactivity.	Intensity
30. Penley & Tomaka (2002) [36]	US	Community	97 (66/31)	21.05 (4.79)	Experimental (lab stressor; cross-sectional trait–emotion associations)	Abbreviated NEO-PI (Costa & McCrae, 1985): 75-item Big Five self-report, 5-point Likert	Immediate post-task self-report of 16 emotions on 9-point Likert; aggregated into Positive and Negative emotion composites (factor-based)	Higher (N) was associated with higher negative emotions. Higher (E) was associated with higher positive emotions and lower negative emotions. Higher (A) was associated with higher positive emotions. Higher (O) was associated with lower negative emotions. Higher (C) was associated with higher positive emotions and lower negative emotions.	Frequency (Positivity/Negativity)

### Study selection and characteristics

The final selection included 30 records, comprising 31 studies and yielding 50 independent samples that met all inclusion criteria, spanning 25 years (1999–2024). Analysis of publication dates indicates a clear upward trend in research output. While early publications were sporadic, the volume of studies increased noticeably between 2014 and 2016, reaching a peak in 2021 with 5 studies (16%). This distribution highlights a steady increase in research attention toward the interaction between personality traits and emotions over the last decade. However, this trajectory reflects the characteristics of literature meeting our specific eligibility criteria and does not necessarily imply a universal shift in the broader research field.

In terms of geographic distribution, the samples were predominantly recruited from Western nations. The United States was the primary source of data, contributing nearly half of the independent samples (21 samples, 42%). European nations comprised a significant share, led by the Netherlands and Belgium (4 samples, 8% each), alongside contributions from Germany, Spain, Norway, and Serbia. Conversely, representation from the non-West remained limited, with only occasional contributions from Asia (China, Japan, and the Philippines) and Venezuela. Regarding sample characteristics, the majority of studies utilized non-clinical community populations (48 samples, 96%), which consisted largely of university students. Consequently, clinical groups were represented by only 2 samples (4%).

Regarding methodological approaches, the review covered various designs, with a notable proportion using longitudinal designs (24 samples, 48%) that employed Experience Sampling Methods (ESM) and daily diary protocols to assess daily emotional fluctuations. Cross-sectional surveys constituted the second largest group (18 samples, 36%), while the remaining 16% (8 samples) employed experimental paradigms involving mood induction or standardized stress tasks. While self-report inventories were ubiquitous, physiological indices such as electrodermal activity and heart rate were utilized in specific studies to provide objective markers of affective intensity and sensitivity. With respect to four dimensions of emotional dynamics, the literature demonstrated a pronounced thematic imbalance. Research attention focused primarily on frequency of valence (22 studies, 70.97%) and intensity (11 studies, 35.48%). Sensitivity and persistence were both underrepresented in the literature, accounting for 4 (12.0%) and 3 studies (9.68%), respectively, indicating a limited empirical understanding of both the threshold and temporal dynamics of emotional responses in relation to personality traits.

## Big five personality traits and emotional characteristics

### Openness to Experience

***Frequency.*** Findings regarding the frequency of positive and negative emotional experiences associated with Openness to Experience (O) are mixed. With respect to negativity, some studies report that higher Openness to Experience (O) is associated with more frequent negative emotions [64,70,71]. In contrast, other studies suggest that (O) is linked to lower levels of negativity [65], including specific emotions such as fear, shame, and anxiety [36,66], or find no significant association between (O) and negative emotions [72]. Regarding positive frequency, the majority of studies indicate that (O) is associated with more frequent experiences of positive emotions [64,65,67–69], with this pattern replicated across cultural contexts [66]. However, one study reported only weak associations [80], whereas another study found no significant relationship between (O) and positive frequency [72].

***Intensity.*** Evidence concerning emotional intensity is inconsistent. For negative emotions, (O) has been linked to lower self-reported intensity [73], yet higher physiological arousal responses (e.g., increased respiratory rate) to negative stimuli [74]. For positivity, some studies suggest that (O) predicts greater intensity of positive emotions [69,73], whereas others report no significant associations with the intensity of either positive or negative emotions [68,73].

***Sensitivity and Persistence.*** (O) was positively associated with heightened aesthetic and social-emotional sensitivity [75] and greater emotional responsiveness to music [76]. No eligible studies examined the association between Openness to Experience and emotional persistence.

### Agreeableness

***Frequency.*** Agreeableness (A) functioned primarily as a predictor of more frequent positive emotions and lower levels of negative emotions, particularly within social contexts. Higher (A) was associated with greater daily positive emotions [72,77], expressed most clearly in more frequent prosocial emotions such as hope and relief [66], happiness [36], and care [67]. Conversely, agreeable individuals tended to report lower overall negativity [71], reflected in reduced levels of socially disruptive emotions including anger [78], irritability [68], and boredom [66].

Importantly, this generally adaptive emotional profile was not uniform but appeared contingent on situational demands and contextual vulnerabilities. In high-threat or competitive environments, the buffering role of (A) was attenuated; for example, higher (A) predicted increased fear during military deployment [81] and elevated negative emotions in competitive academic settings [80]. Cross-cultural findings further suggest that while associations with positive emotions are relatively robust, reductions in negative emotions linked to (A) are less consistent across cultural contexts [79]. Finally, a discrepancy emerged between momentary emotional experience and retrospective evaluation: agreeable individuals reported higher levels of happiness in daily life but lower levels when recalling their experiences retrospectively, indicating potential biases in memory-based emotion judgments [64].

***Intensity.*** Agreeableness (A) was associated with heightened positive emotional responses in interpersonal contexts alongside attenuated expressions of hostility. Across studies, agreeable individuals reported greater intensity of positive emotions during social interactions [73], particularly feelings of pleasantness, pride, and calmness [69]. This generally adaptive profile extended to anger regulation, as (A) was linked to lower anger intensity even under conditions of elevated physiological arousal [78]. In contrast to its buffering effects on hostility, higher (A) predicted heightened reactivity to sadness, characterized by more intense negative emotional reactions during sadness inductions [73].

***Sensitivity and Persistence.*** Although empirical evidence on emotional persistence and sensitivity remains limited, the available findings suggest a profile characterized by relatively rapid emotional recovery alongside heightened responsiveness to environmental and social cues. With respect to persistence, (A) was associated with shorter durations of negative emotional states, such as reduced daily anger persistence, indicating more efficient emotional recovery [78]. In terms of sensitivity, (A) showed a strong association with the Aesthetic and Social Sensitivity factor of the Highly Sensitive Person scale, reflecting heightened attunement to environmental subtleties and interpersonal signals [75]. Complementing these cross-sectional findings, longitudinal evidence indicates that this heightened sensitivity may extend to stress reactivity over time, as increases in (A) were linked to a sharper increase in negative affect in response to daily hassles in adolescents and young adults [82].

### Conscientiousness

***Frequency.*** Conscientiousness (C) is associated with an adaptive emotional profile characterized by more frequent positive emotions and less frequent negative emotions. Multiple studies show that higher (C) predicts more frequent experiences of positive emotions, including happiness, hope, pride, compassion, relaxation, assurance, attentiveness, and determination [36,66,68]. Conversely, (C) is linked to lower levels of negative emotions such as anger [83], shame [81], fear [36], and feelings of hopelessness and disappointment [66]. Notably, Leger et al. [77] demonstrated that this protective effect extends to stressful contexts, with higher (C) predicting reduced negative emotion even on days when stressors occurred. Additionally, retrospective data indicate that higher (C) is associated with lower reporting of both positive and negative emotions over longer recall periods, suggesting a stabilizing or dampening effect on emotional recall rather than momentary experience [64].

***Intensity.*** Findings regarding emotional intensity are more nuanced. Several studies indicate that higher (C) is generally associated with reduced subjective intensity of negative emotional states, including lower negative emotion intensity in individuals with borderline personality disorder [84], and smaller increases in negative emotions following laboratory stress induction [68]. However, this attenuation at the experiential level does not appear to extend to physiological responding. In particular, higher (C) has been linked to greater autonomic activation, reflected in elevated heart rate during exposure to threatening stimuli [74]. With respect to positive emotions, (C) predicts greater emotional intensity in socially meaningful contexts, such as heightened feelings of pride and interpersonal closeness [69].

***Sensitivity and Persistence.*** Evidence regarding emotional sensitivity remains limited. At the trait level, (C) shows a small negative association with sensitivity, suggesting reduced subjective responsiveness to emotional stimuli [75]. In contrast, psychophysiological evidence points to heightened sensory responsivity to negative stimuli, including faster and more frequent electrodermal reactions [85]. Notably, no studies to date have examined the association between (C) and the persistence of emotional responses, highlighting a clear gap in the literature.

### Neuroticism

***Frequency.*** Neuroticism (N) was most consistently associated with an adverse emotional profile, characterized by more frequent negative affect, reduced positive affect, and heightened reactivity to stressors and aversive stimuli. Across studies, higher (N) predicted greater everyday negative affect and lower positive affect, a pattern observed in daily diary and experience-sampling research as well as in retrospective and cross-cultural designs [64,72,77,79]. This pattern was reflected in reports of specific emotions, with higher (N) linked to greater sadness, fear, anger, guilt, shame, self-disgust, nervousness, and distress, alongside lower happiness and reduced positive emotions such as hope, relaxation, and assurance [36,65,66,68,71,80]. Similar associations were observed across both community samples and clinical populations, including individuals with borderline personality disorder, depressive disorders, and autism spectrum conditions [84,86]. Longitudinal evidence further indicates that this pattern extends across time, with higher (N) associated with elevated negative affect across measurement waves, smaller midlife declines, and steeper increases in negative affect in later adulthood [81,87].

***Intensity.*** Evidence on emotional intensity showed a more nuanced pattern, partly depending on the response system being measured. Higher (N) was associated with stronger negative affect intensity in self-report studies, and in some studies it was also linked to stronger positive affect intensity [73], indicating that heightened emotional responding may extend beyond negative affect alone. Physiological evidence further indicated heightened reactivity to aversive stimuli, with higher (N) predicting greater arousal, including elevated skin conductance responses and stronger physiological and subjective arousal during exposure to unpleasant material [74,88].

***Sensitivity and Persistence.*** With respect to persistence, higher (N) was associated with longer duration of negative emotions, reduced reduction of negative emotion when adverse circumstances improved, poorer maintenance of positive emotions, and slower physiological habituation to aversive stimuli [37,38,88]. With respect to sensitivity, Neuroticism was linked to stronger affective responses to stressors and hassles, including greater stressor-related negative affect, stronger daily stress reactivity, and larger momentary increases in negative affect in response to everyday difficulties [17,77,82,89].

### Extraversion

***Frequency.*** Extraversion was associated with a predominantly positive emotional profile, characterized by more frequent positive emotions and, in many contexts, fewer negative emotions. Evidence on primary emotion systems indicated that higher (E) was associated with SEEK, PLAY, and ANGER and inversely associated with FEAR [67]. Across multiple other studies, higher (E) predicted greater happiness, pride, enthusiasm, excitement, interest, and activity, and was often linked to lower fear, guilt, nervousness, and self-disgust [36,65,68,80]. Similar patterns emerged in daily and retrospective reports: higher (E) predicted greater daily positive affect, lower daily negative affect, reduced negative affect on days when stressors occurred, and higher same-day and 2-week retrospective happiness alongside lower sadness and fear [64,77]. However, associations with negative affect were not consistently observed. Some studies reported weaker or non-significant links between (E) and negative affect [72,79], and in achievement contexts (E) predicted both positive emotions (e.g., pride) and negative emotions such as anxiety, shame, and disappointment [66]. Longitudinal evidence further showed that although (E) was associated with higher overall levels of positive affect, it did not predict systematic changes in affective levels over time [87].

***Intensity.*** The available evidence generally suggested stronger positive emotion intensity among individuals higher in Extraversion. Self-report studies found that higher (E) predicted greater positive emotion intensity [38,73], whereas physiological findings were less consistent, with context-specific associations indicating lower heart-rate responses to tension and violence–fear scenes [74]. Evidence on emotional persistence was more limited but provides preliminary support for a link between (E) and more sustained positive emotional experiences. This pattern is reflected both in directly assessed emotional duration [38] and indirectly in a greater tendency to savor positive experiences, which may prolong positive affect [37].

***Sensitivity and Persistence.*** Research addressing emotional sensitivity was especially scarce. Only one study examined this domain using music stimuli and found that higher Extraversion was associated with greater emotional responsiveness to music and fully mediated the relationship between autistic traits and music-evoked emotional responsiveness [76].

## Discussion

This systematic review examined how the Big Five personality traits are characterized by different dimensions of emotional reactivity, including intensity, sensitivity, persistence, and the tendencies toward positive or negative emotional experiences. By synthesizing evidence across the available literature, it aimed to characterize the distinct emotional reactivity profiles associated with each personality trait. Overall, the findings indicate that each trait exhibits a unique pattern of emotional reactivity. Neuroticism showed the most consistently maladaptive associations across all dimensions, whereas the other traits demonstrated varying degrees of consistency across emotional reactivity dimensions. These results suggest that personality traits within the Big Five influence specific aspects of emotional reactivity rather than reflecting a single, uniform pattern.

In order to provide a clear and systematic discussion of the findings, we first summarize the emotional reactivity profile of each Big Five personality trait, interpret the observed patterns in relation to existing theories and potential explanatory mechanisms, consider relevant contextual and individual influences, and discuss the implications for mental health. Finally, we integrate these trait-specific patterns to explore their broader implications for understanding the relationship between the Big Five personality traits and emotional reactivity.

### Openness to experience

Although the most consistent finding is more frequent positive emotional experiences, Openness appears to be less defined by uniform increases or decreases in specific emotional states. Instead, the heterogeneity of this emotional profile may point to the inherent complexity of its emotional characteristics. This pattern may partly reflect what earlier models describe as psychological complexity, conceptualizing Openness as a core driver of the breadth and richness of psychological life [90] and as a dispositional tendency toward exploration of novel experiences [91]. This exploratory orientation may foster frequent positive affect through engagement in creative and novel activities, while simultaneously increasing exposure to a broader range of emotional experiences [92].

Notably, the findings also point to a divergence between objective and subjective emotional responses. In particular, Openness has been linked to lower self-reported intensity of negative affect [73], yet higher physiological arousal responses to emotional stimuli, particularly sadness [74]. One possible explanation is that individuals higher in Openness do not exhibit reduced emotional reactivity at the physiological level, but rather differ in how these experiences are appraised and regulated. While heightened physiological arousal suggests strong responsiveness to emotional stimuli, individuals high in Openness may adopt a more curious, exploratory, or accepting stance toward these experiences, thereby reducing their perceived intensity or distress. Converging evidence from stress-related contexts further supports this interpretation. Higher Openness has been associated with lower cardiovascular reactivity and more efficient autonomic recovery in response to repeated stress [93], indicating a more adaptive pattern of physiological regulation. At the cognitive level, individuals high in Openness also tend to appraise daily stressors as less severe [77], which may attenuate subjective distress. In addition, they are more likely to engage in adaptive regulation strategies, such as problem solving or mindfulness, rather than maladaptive strategies like avoidance or suppression [31]. Together, these findings suggest that individuals high in Openness may experience strong emotional activation while simultaneously engaging cognitive and regulatory processes that buffer its subjective impact.

The emotional profile associated with Openness to Experience may help explain its clinical implications. While largely unrelated to externalizing problems, Openness appears to show a modest protective association with internalizing psychopathology, including depression and anxiety [94]. One possible explanation lies in its emotional pattern, as the most consistent finding in the present synthesis is a tendency toward more frequent positive emotional experiences. This positive emotionality, combined with a greater tendency to appraise stressors as less threatening and to engage in adaptive regulation strategies, may help buffer the subjective impact of distress. In addition, individuals high in Openness may be more likely to engage with emotional experiences in a flexible and exploratory manner rather than avoiding them, further supporting resilience in the face of everyday stressors. However, this protective pattern is not equally evident. Given that Openness is associated with heightened experiential sensitivity, its benefits may depend on contextual conditions. In persistently adverse environments, this heightened receptivity may amplify emotional reactions to negative experiences, thereby increasing emotional distress and vulnerability rather than promoting resilience [95].

### Agreeableness

The current synthesis suggests that Agreeableness reflects a strong orientation toward interpersonal attunement and social harmony. This affiliative tendency appears to shape a distinct emotional pattern characterized by heightened positive emotionality and reduced socially disruptive negative states. Such findings align with foundational models that conceptualize Agreeableness primarily as a prosocial and communal disposition [91,96]. Through empathic sensitivity and concern for others, highly agreeable individuals may be more likely to cultivate affiliative emotions and maintain positive emotional states during interpersonal interactions. This orientation may also help regulate antagonistic reactions: when confronted with hostility, agreeable individuals tend to spontaneously recruit prosocial thoughts and exert greater behavioral control to suppress aggressive impulses [78,97]. In addition, this adaptive emotional style may partly reflect the greater use of adaptive emotion-regulation strategies among agreeable individuals, including cognitive reappraisal, problem solving, or mindfulness [31]. Together, these tendencies likely support the maintenance of positive interpersonal dynamics and harmonious social exchanges.

However, the findings also suggest a paradox in the interpersonal sensitivity associated with Agreeableness. While the trait promotes social harmony, its heightened attunement to others may also increase vulnerability to emotional contagion. Individuals higher in Agreeableness show stronger negative emotional reactivity when exposed to others’ suffering or sad stimuli [73]. This heightened sensitivity may extend beyond negative contexts, as even during personal positive events, highly agreeable individuals experience elevated negative affect, such as guilt or ambivalence, reflecting concern that their success could negatively affect others [69].

Importantly, the emotional profile of Agreeableness should be interpreted with caution. Although experimental evidence indicates that Agreeableness is linked to heightened state reactivity in response to negative stimuli, such as sadness, it does not appear to be consistently associated with baseline emotional states [73]. This suggests that its distinctive emotional expression may be emerging primarily in response to emotionally salient or distress-related stimuli, although further research is needed to clarify these patterns. Moreover, the predominantly adaptive emotional profile of Agreeableness may be moderated by several individual factors. Consistent with the behavioral concordance model, when agreeable individuals rely on trait-incongruent avoidant coping strategies, they report lower positive affect [70]. In addition, the protective role of Agreeableness in reducing externalizing tendencies also depends on other personality traits. Agreeable individuals typically show reduced aggression following exposure to aggression-related cues, partly through the activation of prosocial thoughts [97]; however, this protective effect is attenuated among those low in Conscientiousness [83].

Overall, the emotional profile associated with Agreeableness may help explain its distinct associations with mental health outcomes. Highly agreeable individuals demonstrate a modest protective association against internalizing disorders, including depression and anxiety [98]. This resilience is partially mediated by their capacity to experience higher general intensity of positive emotions [73], which may help counteract the accumulation of daily depressive symptoms. Conversely, low Agreeableness is conceptualized as antagonism, a robust predictor of externalizing problems, such as aggression and antisocial behavior [99,100]. This vulnerability mainly corresponds to an emotional system characterized by elevated intensities of anger and interpersonal hostility [78].

### Conscientiousness

This review characterizes Conscientiousness as reflecting an emotionally adaptive and well-regulated profile. Individuals high in Conscientiousness tend to experience more frequent positive and fewer negative emotions. This pattern may reflect an accompanying adaptive cognitive style, whereby Conscientiousness is closely linked to more effective emotion regulation, largely due to its foundation in strong self-control [83] and its goal-directed orientation, characterized by planning, organization, and thinking before acting [101]. These regulatory capacities allow highly conscientious individuals to down-regulate negative feelings while maintaining positive emotional states when challenges arise. In addition, their goal-directed approach likely encourages a pragmatic and controlled response to stressors rather than impulsive reactions.

Beyond the frequency of valenced experiences, this synthesis also observes a divergence whereby higher Conscientiousness is associated with attenuated subjective intensity of negative emotions, while physiological studies indicate stronger stress-related activation. This apparent paradox may be accounted for by prior research showing that Conscientiousness predicts greater recovery from negative stimuli but not reduced initial reactivity [102]. In other words, the initial response is reflected in physiological evidence, including higher cortisol levels and greater amygdala–insula activation during social-evaluative stress [103], as well as increased cardiac effort during incentive tasks [104]. Over time, however, the superior self-control and emotion regulation capacities associated with Conscientiousness may facilitate more effective modulation of these responses, resulting in a dampened subjective intensity of negative emotions despite strong initial physiological mobilization.

The relationship between Conscientiousness and emotional experience is also influenced by both situational context and coping mechanisms. First, evidence shows that the protective association between Conscientiousness and negative affect becomes particularly pronounced on days when individuals encounter multiple stressors, whereas the effect is weaker on stress-free days [77]. This pattern suggests that Conscientiousness may function as a regulatory resource activated when environmental demands increase. Second, coping mechanisms may help explain how this regulatory advantage operates. Individuals low in Conscientiousness tend to experience substantially higher negative affect when relying on avoidance strategies like wishful thinking or emotional constraint [70]. In contrast, those higher in Conscientiousness are more likely to adopt approach-oriented coping, including problem solving and positive reappraisal. These strategies partially mediate the relationship between Conscientiousness and emotional outcomes, contributing to both increased positive affect and reduced negative affect [105].

These emotional patterns have important implications for mental health. Conscientiousness appears to protect psychological well-being through its role in promoting emotional stability and effective regulation. Individuals with lower levels of Conscientiousness have been linked to internalizing disorders such as depression and generalized anxiety, particularly through facets reflecting low self-efficacy, poor competence, and high distractibility [106]. Over time, these individuals may experience functional difficulties in daily life that foster negative self-perceptions and persistent negative affect, while also directly increasing the risk of externalizing problems through heightened impulsivity and short-term reward-seeking tendencies [107]. Conversely, the strong effortful control associated with high Conscientiousness enables individuals to recover more effectively from negative emotional stimuli, preventing temporary distress from developing into chronic conditions [102]. Conscientiousness may also shape how stressful experiences are processed; for instance, highly conscientious individuals may show a weaker association between social withdrawal and depressive symptoms by shifting attention away from negative emotions associated with isolation [108].

### Neuroticism

Neuroticism showed the most consistently maladaptive emotional profile in the present review. Across studies, higher Neuroticism was associated with more frequent negative affect, lower positive affect, greater emotional intensity, slower emotional recovery, and heightened sensitivity to stressors. This pattern is consistent with long-standing trait accounts that conceptualize Neuroticism as a dispositional tendency toward distress, threat sensitivity, and vulnerability to stress rather than as a marker of any single emotion or disorder (for reviews, see [109]). Importantly, although the emotional characteristics of Neuroticism were predominantly associated with heightened negative affect, limited evidence suggests that when positive emotions do occur, they may also be experienced more intensely but maintained less effectively [37,73]. This points to a more complex emotional profile than earlier conceptualizations that focused primarily on negative emotions alone.

However, several factors may moderate the expression of the emotional profile associated with Neuroticism. One potential moderator is social context. For example, in a study of individuals on the autism spectrum, those higher in Neuroticism reported stronger negative affect when interacting with less familiar people compared with when they were alone or with familiar others [86]. This pattern suggests that individuals high in Neuroticism may be particularly susceptible to increases in negative affect in socially unfamiliar situations, although further research is needed to determine whether this effect generalizes beyond this population. Individual characteristics such as age may also influence the association between Neuroticism and emotional style, although the available evidence is mixed. Age-related differences have been observed in Neuroticism-related emotional responses to everyday stressors, with some studies reporting stronger effects in older adults and others suggesting that the association may be more pronounced in young adulthood [17,82].

The predominantly maladaptive emotional profile associated with Neuroticism may partly stem from the coping strategies commonly linked to this trait. Individuals higher in Neuroticism tend to rely more on maladaptive regulation strategies, including suppression, rumination, and worry, while showing lower use of more adaptive strategies such as reappraisal and mindfulness [31]. In addition, Neuroticism is most consistently associated with avoidant coping, which typically involves withdrawal, disengagement, and denial rather than direct engagement with stressors [110]. By limiting opportunities for constructive problem engagement and emotional reappraisal, these coping tendencies may sustain or amplify negative emotional states.

The identified maladaptive emotional profile may help explain why Neuroticism has long been associated with a wide range of psychological difficulties. Higher Neuroticism has consistently been linked to internalizing psychopathology, particularly mood and anxiety disorders, and has also been associated with externalizing problems, including substance misuse and antisocial behavior [111,112]. More recent work further conceptualizes high Neuroticism as a transdiagnostic risk factor that cuts across multiple dimensions of psychopathology rather than serving as a marker of any single diagnostic category [113,114]. The present review contributes to this perspective by identifying a plausible emotional pathway through which Neuroticism may increase vulnerability to mental-health difficulties, characterized by an emotional system in which distress is more easily activated, less effectively regulated, and slower to resolve.

### Extraversion

Extraversion was associated with a consistently positive emotional profile in the present review, characterized more by the reliable presence of positive affect than by the consistent absence of negative affect. Across studies, higher Extraversion was linked to greater happiness, enthusiasm, interest, pride, and activity, with similar patterns emerging in daily-life and retrospective reports of higher positive affect and, in some cases, lower sadness and fear. Although this association between Extraversion and positive affect appears relatively consistent, even across cultures, its association with negative affect is generally weaker. Overall, the findings appear generally consistent with trait accounts that conceptualize Extraversion as a dispositional tendency toward positive emotionality, reward sensitivity, and energetic engagement with pleasurable experiences [91]. However, this positive emotional pattern does not appear equally strong across all contexts. For example, in achievement settings, Extraversion was associated not only with pride but also with anxiety, shame, and disappointment [66]. This suggests that when performance and evaluation become salient, Extraversion may reflect stronger motivational engagement rather than simply reduced distress.

One possible explanation for the more adaptive emotional profile associated with Extraversion lies in the way individuals high in this trait cope with life challenges. In particular, individuals higher in Extraversion tend to employ more adaptive regulatory strategies, including reappraisal, problem solving, and mindfulness (see [31], for a review). Consistent with their approach-oriented style, they are also more likely to make use of coping strategies that involve actively addressing stressors and mitigating their emotional impact. In line with this, evidence suggests that approach coping, particularly emotional support–seeking coping, may help account for the positive emotion facets of Extraversion [70,115].

The favorable pattern in emotional style may help explain why Extraversion is often linked to greater life satisfaction and broader well-being, as well as an inverse association with internalizing psychopathology, including depression and anxiety [107,116]. Frequent positive affect, stronger reward responsiveness, and better maintenance of positive emotional states may help interrupt stress-related negative spirals and support emotional recovery in everyday life. However, this implication should not be interpreted as indicating uniform protection associated with Extraversion. Negative emotional outcomes can still emerge, particularly in evaluative or goal-blocking contexts, where the same approach-oriented engagement that supports positive emotion may also increase exposure to frustration, disappointment, or performance-related anxiety [66,67].

### Broader implications

First, the present review suggests that the associations between the Big Five personality traits and emotional reactivity are best understood as multidimensional. By synthesizing evidence across multiple dimensions of emotional reactivity, the present review shows that each trait is characterized by a distinct reactivity profile rather than a uniformly adaptive or maladaptive emotional profile. More broadly, this multidimensional perspective supports ongoing efforts in personality theory to move beyond broad descriptive trait taxonomies toward a deeper understanding of the processes through which personality influences behavior [109]. As Roberts (2009) noted, progress in personality science depends not only on identifying broad trait dimensions but also on elucidating the mechanisms through which these traits give rise to thoughts, feelings, and behaviors [29]. In this context, the present review contributes by showing that broad personality traits can be characterized in terms of distinct patterns of emotional responding, including the tendency to experience positive or negative emotions, how strongly they are experienced, how readily emotions are elicited, and how long they persist. Although these dimensions do not constitute the personality traits themselves, they provide a more fine-grained characterization of the emotional processes through which personality traits may be expressed in everyday emotional functioning.

Second, in terms of measurement, the present synthesis suggests that subjective and physiological measures of emotional reactivity do not always converge. This divergence was most evident for Openness and Conscientiousness, both of which showed lower self-reported intensity of negative emotions despite heightened physiological arousal. A similar pattern was also observed for Agreeableness, although the available evidence was more limited. By contrast, Neuroticism showed convergence across measurement modalities, with both subjective and physiological indicators suggesting heightened emotional reactivity. Together, these findings indicate that emotional reactivity cannot be fully characterized using a single assessment method, as self-report and physiological measures may capture different aspects of emotional responding. At the same time, methodological heterogeneity, including differences in emotion induction paradigms, assessment contexts, and measurement approaches, likely contributed to inconsistencies across studies.

Third, with respect to mental health, previous research has identified emotional reactivity as a transdiagnostic vulnerability factor across psychiatric conditions [54]. The present review extends this perspective by suggesting that multidimensional emotional reactivity may represent one possible pathway through which personality traits contribute to individual differences in mental health risk. Rather than treating personality traits solely as broad risk factors for psychopathology, the present findings suggest that their characteristic patterns of emotional reactivity may help explain why some personality traits confer greater vulnerability whereas others appear to be protective. However, this interpretation remains tentative because the available evidence does not directly examine whether emotional reactivity mediates the relationship between personality traits and mental health outcomes. Future longitudinal studies using mediation or other process-oriented designs are needed to test this possibility.

### Limitations and future research

This review synthesizes a substantial body of literature but is subject to several limitations that may influence the interpretation of its findings. First, heterogeneity in the conceptualization and measurement of emotional reactivity limits direct comparability across primary studies. Although reclassifying these diverse indicators into four predefined domains was necessary for synthesis, this process inevitably introduces some degree of subjectivity. Moreover, this four-domain framework captures only a partial view of emotional functioning, as other important facets (e.g., emotional instability or inertia) fall beyond the scope of the present review. Second, systematic imbalances in the primary literature constrain the depth and robustness of the resulting emotional profiles. Research has disproportionately focused on Neuroticism and Extraversion, primarily examining emotional frequency and intensity. In contrast, Openness, Agreeableness, and Conscientiousness, as well as key dimensions such as sensitivity and persistence, remain comparatively understudied. Third, although this review integrates both subjective and physiological evidence, the broader literature’s heavy reliance on self-report measures provides only a partial account of emotional reactivity. This limitation is underscored by the divergence observed between subjective and physiological response systems, particularly for traits such as Openness and Conscientiousness. Fourth, a formal assessment of reporting bias was not conducted due to the heterogeneity of study designs, outcomes, and synthesis methods. Although the use of multiple databases and broad search terms was intended to reduce the risk of missing relevant studies, reporting bias cannot be ruled out. Finally, the overrepresentation of Western, non-clinical samples limits the generalizability of the findings to more diverse and clinical populations.

The certainty of the evidence should therefore be interpreted as variable across traits and emotional domains. Confidence is higher for patterns that were supported consistently across multiple studies, particularly the association between Neuroticism and negative emotionality and between Extraversion and positive emotionality. In contrast, conclusions regarding Openness, Agreeableness, Conscientiousness, and less frequently studied domains such as sensitivity and persistence remain more tentative because of limited evidence, measurement variability, and reliance on predominantly observational and self-report designs.

To address these limitations, future research should prioritize multidimensional operationalizations of emotional processes in affective and clinical research, ensuring balanced coverage across personality traits and greater attention to underexamined dimensions such as sensitivity and persistence. In particular, future studies should clearly specify which dimensions of emotional reactivity are being assessed, as greater conceptual precision in measurement is essential for accurately identifying and comparing emotional processes across studies. In addition, integrating behavioral and physiological measures within longitudinal designs, and extending research to more diverse cross-cultural and clinical samples, will be critical for clarifying how personality traits and emotional dynamics interact over time. Furthermore, future research should examine how personality traits and their associated emotional profiles relate to downstream mental health and behavioral outcomes, thereby informing the development of more targeted prevention and intervention strategies.

Beyond the Big Five, future research may also examine whether the multidimensional framework of emotional reactivity can be extended to alternative models of personality. For example, Reinforcement Sensitivity Theory (RST), which conceptualizes personality in terms of individual differences in sensitivity to reward, punishment, and threat, may provide a theoretically relevant framework for extending this line of research. The observed conceptual overlap between RST and the Big Five [34] further highlights the value of examining emotional reactivity across multiple personality models. Comparing emotional reactivity profiles across different personality frameworks may help determine whether similar emotional mechanisms underlie distinct models of personality or whether each framework captures unique aspects of emotional experiences.

## Conclusion

This systematic review provides a comprehensive synthesis of how the Big Five personality traits are associated with distinct patterns of emotional functioning across core domains of emotion reactivity, including intensity, sensitivity, persistence, as well as the frequency of positive and negative emotional experiences. The findings indicate that each trait is characterized by a unique emotional profile, reflecting not only differences in valence but also in how emotional responses are triggered and unfold over time.

In particular, Neuroticism was consistently associated with a maladaptive emotional profile marked by heightened negative affect, increased sensitivity to stress, and prolonged emotional responses, whereas Extraversion and Conscientiousness were linked to more adaptive patterns characterized by greater positive emotionality and more effective regulation. Openness to Experience was associated with a more complex and differentiated emotional profile, while Agreeableness reflected an interpersonal emotional style marked by prosocial emotionality alongside context-dependent vulnerability to emotional contagion.

By disentangling multiple components of emotion reactivity across traits, this review advances a more nuanced and multidimensional understanding of personality-emotion relationships. Importantly, the findings suggest that emotion reactivity may represent a key mechanism through which personality traits shape mental health outcomes. These insights contribute to refining theoretical models of personality and highlight the value of integrating multidimensional emotional processes in future affective and mental health research.

## Supporting information

S1 Checklist PRISMA 2020 checklist. (DOCX)

S1 Table JBI Quality Assessment results. (XLSX)
